# Anemia and its associated factor among adolescent school girls in GODEY and DEGEHABUR council Somali region, eastern Ethiopia

**DOI:** 10.1186/s40795-022-00548-1

**Published:** 2022-06-14

**Authors:** Abdirahman Ahmed, Abdulkarim Mohammed

**Affiliations:** Department of Human Nutrition, Jigjiga University, Jigjiga, Ethiopia

**Keywords:** Adolescent girls, Anemia, Dietary diversity, Menstruation

## Abstract

A cross-sectional study was conducted in a higher secondary school in Godey and Degehabur council to estimate the prevalence of anaemia among school-going adolescent girls and to identify the associated factors. Haemoglobin levels were assessed directly in the school. Data related to socio-demographic, socio-economic characteristics, dietary habits, past health status and anaemia related knowledge among adolescents’ girls were collected by interview method and analyzed with the help of SPSS version 25. The prevalence of anaemia was found to be 31.5% among adolescent girls. Family size ≥5 people and lack of anaemia knowledge were independent predictors of anaemia. Therefore, the present study emphasizes the need for development of health and nutrition education strategies to improve dietary habits, family planning, and intermittent iron-folic acid supplementation with intensifying adolescent anemia prophylaxis programs.

## Background

### Introduction

Anaemia is a global public health problem affecting both developing and developed countries with major consequences for human health as well as social and economic development. It occurs at all stages of the life cycle but is more prevalent in pregnant women and young children [1, 2]. According to the 2016 national micronutrient survey report, 18% of Ethiopian women aged 15–49 are anaemic and are considered a mild public health problem. However, women in the Somali, Gambella, and Afar regions have a relatively high prevalence of anaemia (34.8, 26.7, and 26.2% respectively) compared to the national average. An adolescent is a period of transition between childhood and adulthood is a significant period of human growth and maturation. Being a period of the growth spurt, an exceptionally rapid rate of growth occurs with unique change during this phase of life. Anaemia in adolescent girls affects their physical work capacity and reproductive physiology and work productivity of adults [3, 4]. Although anaemia has been recognized as a public health problem for many years, little progress has been reported and the global prevalence of anaemia remains unacceptably high. WHO and UNICEF, therefore, reemphasize the urgent need to combat anaemia and stress the importance of recognizing its multi-factorial aetiology for developing effective control programmes [5]. With this background, this study has been undertaken to estimate the prevalence of anaemia and associated factor among adolescent school girls in Godey and the Degehabur council Somali region (a region known for food insecurity thus high rate of anemia).

## Materials and method

### Study design, area and period

A school-based cross-sectional study was conducted from February–March, 2019 among adolescent girls in Godey and Degehabur council, Somalia Region.

Gode town is the capital of Shebelle Zone and it is one of six self-administrated city council. Gode is almost 600KM away from the capital of Somali Regional State Jigjiga and it is the town established along with Shebelle River. It has about 10 Kebeles with one general hospital and two health center. The weather is condition is desert.

Degahbur town is the capital city of jarar Zone and it is one of six self-administrated councils. Degahbur is situated about 160 km south east of the regional capital, Jigjiga. The activities of Degahbur City Administration are limited to the ten urban Kebeles (smallest administration units) in Degahbur town. The town has one general public hospital, one health center and six health posts that provide services to the public.

Gode and Degahbur are selected for this particular title because most of the study conducted in Somali region is around Jijiga and there is little study done around other parts of the region. Specifically, concerning this topic, there is no research done so far. Therefore, it is better to fill this and other gaps.

### Inclusion and exclusion criteria

Adolescent girls of age between 15 and 19 years who were willing to participate in study and gave consent for the same and who were present on the day of visit in the school are included in the study while those adolescent girls who are not willing to participate or not giving consent were excluded from the study.

### Sample size determination

The sample size was determined using a single population proportion formula. By the following assumptions: The level of confidence (α) is taken to be 95% (Z1-α /2 = 1.96); the margin of error (d) is taken to be 5% [0.05]. The proportion (p) of adolescents’ girls who were anemic was 32%, according to the study conducted in Babile (13) and with the design effect of 1.5 and 5% a none respondent rate. The final sample size 372 was determined by Cochran’s formula for calculating sample size when population size is finite.

### Sampling techniques

A multi-stage sampling technique was employed primary; two councils (Godey and Degarbor) from two different zones (Shabelle and Jarar) of the Somali region were purposively selected and out of the total 6 public high schools, two were selected using the lottery method. Then, the number of students from each school was proportionally allocated, the female students to include the study were determined by a systematic random sampling method using a student’s registrations book as a sampling fram**e.**

### Data collection method

A semi-structured questionnaire was used to obtain information related to socio-demographic, socio-economic characteristics, past health status and anemia related knowledge among adolescents’ girls living in Godey and Degehabur council Somali region, eastern Ethiopia.

### Dietary characteristic of adolescent girls

An individual 24 hrs. Recall method as recommended in FAO (Food and Agriculture Organization), 2010 and 2016 guideline to collect individual dietary diversity was followed to collect dietary data. Participating girls were asked to report any food and drinks consumed 24 hrs. Preceding the survey. The dietary diversity was calculated after the food items were grouped into 9 categories: (i) starchy staples (ii) Dark green leafy vegetables (iii) Other fruits and vegetables (iv) organ meat (v) flesh meat (vi) eggs (vii) legumes and nut (viii) fish and (ix) milk and milk product.

### Hemoglobin measurement

Adolescent haemoglobin status was measured by using a portable battery-operated photometer (HemoCue hg /301 + Analyser). The capillary blood sample was taken by pricking the tip of the finger in an aseptic way. After rubbing the fingertip with sterile cotton, (immersed in alcohol) a 10 μl blood sample was collected by finger pricking with a sterile disposable lancet and the second blood drop was taken for hemoglobin measurement. The Result was read within one minute. The photometer was calibrated before every session using a provided standard. Hemoglobin level determination was done by trained laboratory technicians working out of the council. Anemia status of adolescent girls was assessed using the WHO (World Health Organization) classification. An individual adolescent girl was considered anemic if the Hb value was below 12.0 g/dL. Girls having anemia were further categorized into different grades such as mild (10–12 g/dL), moderate (7–9.9 g/dL) and severe (< 7.0 g/dl) [14].

### Statistical analyses

Statistical analysis was conducted using SPSS software version 25. Tables, graphs, means, and frequencies were used to present descriptive results. Odds Ratio (OR) was performed to test the association b/n haemoglobin and independent variable. A stepwise binary logistic regression model was applied to test the association between anemia and socio-demographic, socioeconomic, dietary diversity, past medical history, and anemia knowledge. *P* < 0.05 was considered to determine statistical significance.

## Result

### Socio-demographic and economic characteristics of adolescent school girls

A total of 372 school adolescents participated in this study with a response rate of 100%. The mean age with a standard deviation of the adolescent girls was 17.8(±1.2) years. The majority of the participants (98.7%) were Somali followed by Amhara (1.3%). The majority (97.3%) of the respondents were not married. About half of the participants (53.8%) were from a household with a family size greater than five. A summary of the socio-demographic and socio-economic characteristics of the present study participants is indicated in Table 1.

**Table 1:** Socio-economic and demographic characteristics of adolescent school girls in Godey and Degehabur

Variables	n (%)
Age of adolescent	
17–19 15–17	308(82.8%) 64(17.2%)
Religion	
Muslim Orthodox	367(98.7%) 5(1.3%)
Ethnicity	
Amhara Somali	5(1.3%) 367(98.7%)
Marital status	
Married Not married	10(2.7%) 362(97.3%)
Family size	
< 5 > 5	172(46.2%) 200(53.8%)
Family’s Monthly Income	
< 1500(15USD) > 1500	5(1.1%) 368(98.9%)
Mother Education	
Illiterate Informal Education Formal Education	251(67.5%) 9(2.4%) 112(30.1%)

### Anemia prevalence among adolescent school girls

**Fig. 1:**
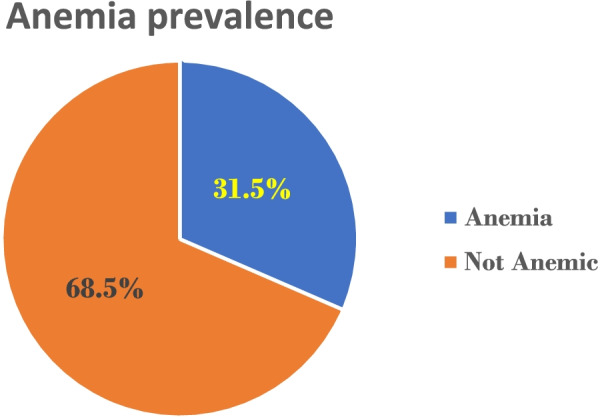
Anemia prevalence among adolescent school girls

The haemoglobin concentration was in the range of 7.3 g/dl to 18.1 g/dl, with a mean value of 12.3 ± 1.3 g/dl. The overall prevalence of anemia was 31.5% in the present study. Out of the total samples, 25.5 and 6% were mildly and moderately anemic, respectively (Fig. 1).

### Knowledge of anemia among adolescent school girls

In the present study, out of 372 adolescent girls, (52.7%) responded that they have heard about anemia. The main sources of information were from health professionals (51%) and family members (33.2%). Half of the perticpants (53.2%) knew that anemia is a health problem. Out of girls who have had about anemia, (74.5%) responded that poor diet is the only cause for anemia and (15.8%) participants didn’t know the answer. In addition, (75%) of the girls having prior knowledge about anemia, answered that tiredness/body weakness is the only manifestation of anemia and (4%) answered anemia manifests shortness of breathing while (2.7%) answered anemia manifestation is difficult to learn. Fifty-six (15%) told anemia impacts only growth and development and (81.5%) participants did not know the answer. Regarding anemia prevention and intervention, out of 372 girls, (17.5%) told green leaf vegetable is the only source of iron-rich food and (8.3%) girls answered meat and poultry are rich of iron while (72%) girls did not know the answer. (Table 2).

**Table 2:** Knowledge of anemia among adolescent school girls in Godey and Degehabur Council

Variables	n(%)
Know/heard about anemia	
**Yes** No	196(52.7%) 176(47.3%)
Source of information	
Teachers Health provisional Family members Media Friends	11(4.1%) 100(51.0) 65(33.2%) 3(1.5%) 20(10.2%)
Is anemia a health problem?	
Yes No	198(53.2%) 172(56.8%)
What are the causes of anemia?	
Poor diet Worm infestation Menstruation Bleeding from wound Pregnancy Don’t know	146(74.5%) 9(4.6%) 1(0.5%) 5(2.5%) 4(2%) 31(15.8)
How do you know if you have anemic?	
Tiredness/body weakness Shortness of breathing Difficult to learn Don’t know	147(75%) 8(4%) 4(2%) 37(19%)
What are the consequences of anaemia?	
Impact on growth and development poor school performance Decreased wok capacity Don’t know	56(15%) 10(2.7%) 3(0.8%) 303(91.5%)
Which one of the following is Iron-rich food?	
Green leaf vegetable Legumes Meat and poultry Don’t know	65(17.5%) 8(2.2%) 31(8.3%) 268 (72%)

### Dietary characteristic of adolescent school girls

The mean dietary diversity of the present study participants was 4.2 ± 0.8 with a range of 2 to 8, out of the 9 points.

All of the participants (100%) ate cereal-based foods. In addition, (52.2%) of the adolescents ate legumes 24 hours preceding the interview. Furthermore, 99.7, 79.3, 87.9, 3.2, 1.1, and 1.6% of adolescent girls ate vegetables, fruit, fresh meat, organ meat, fish, and eggs; respectively (Table 3).

**Table 3:** Dietary characteristic of adolescent school girl

Variables	n (%)
Cereals and tubers	
Yes	372(100%)
Legumes and nuts	
Yes No	194(52.2%) 178(42.8%)
Vegetables	
Yes No	371(99.7%) 1(0.3%)
Fruits	
Yes No	295(79.3%) 77(20.7%)
Organ meat	
Yes No	12(3.2%) 360(96.8%)
Flesh meat	
Yes No	327(87.9%) 45(12.1%)
Fish	
Yes No	4(1.1%) 368(98.9%)
Eggs	
Yes No	6(1.6%) 366(98.4%)
Milk and Milk product	
Yes No	15(4%) 357(96%)

The present study found that (3.8%) of girls fulfilled the minimum recommended dietary diversity according (MDD-W ≥ 5 food groups). In addition, most adolescent girls had low consumption of animal source foods particularly organ meat, fish, eggs, and milk.

### Factors associated to anemia among adolescent school girls

In a bivariate logistic regression model variable with significant associations were identified. Finally, those variables which have association in bivariate models were taken to multivariate logistic regression to compare the independent associations for solving cofounding effects of the variables. In multivariate logistic regression; family size [(AOR = 1.80), CI: (1.14, 2.85)] and adolescent girl who never heard anemia1.6 [AOR = 1.62), CI (1.01,2.59)] were significantly associated with anemia (Table 4).

**Table 4:** Factors associated with anemia among adolescent school girls in Godey and Degehabur council

Variables	Response	n	%	COR(95% CI)	AOR(95% CI)
**Age**	17–19	308	82.8%	1	1
	15–16	64	17.2%	2.24(1.1,4.3)	1.45(0.6, 3.0)
**Family size**	< 5 people	172	46.2%	1	1
	> 5 people	200	53.8%	1.56 (1.0, 2.4)	1.8(1.1, 2.8)
**Family-monthly Income**	< 1500(15USD)	5	1.3%	1	1
	> 1500	367	98.7%	0.72(0.7,7.0)	0.6(0.06, 6.16)
**Menstrual pattern**	Regular	56	15.1%	1	1
	Irregular	316	84.9%	2.0(0.2,0.97)	2.0(0.23, 1.02)
**Duration of menstruation (days)**	< 5 days	139	37.4%	1	1
	> 5 days	233	62.6%	1.61(1.0,2.5)	0.9(0.53, 1.55)
**Malaria history last 14 days**	Negative	313	84.1%	1	1
	Positive	59	15.9%	1.2(0.5,2.61)	1.29(0.55, 3.0)
**Minimum dietary diversity**	< 5 Food group	358	96.2%	1.67(0.5,4.9)	1.76(0.5, 5.42)
	> 5 Food Group	14	3.8%	1	1
**Knowledge of anemia**	Heard anemia	196	52.7%	1	1
	Never heard anaemia	176	47.3%	1.78(1.1,2.7)	1.62 (1.0,2.59)

*COR* Crude odds ratio, *AOR*Adjusted odds ratio

## Discussion

Anaemia is a major public health concern for adolescent girls in developing countries, with negative implications for growth, birth outcomes, and long-term health. This study was designed to estimate the prevalence of anemia among adolescent girls in the Somalia region (a region known for food insecurity thus high rate of anemia). Also, the study attempted to identify factors associated with low haemoglobin concentration. The haemoglobin concentration was in the range of 7.3 g/dl to 18.1 g/dl, with a mean value of 12.3 ± 1.3 g/dl. Anemia was prevalent in 31.5% of the study participants.

Anemia prevalence in this study found was 31.5%, which this higher than the national average of about (18%) (Ethiopian notational micronutrient survey (2016), and (24%) (EDHS, (2016). but lower than the ENMNS (2016) finding in the Somali region which showed (34.8%). however, approximately similar to the report of the study in Babile District, Eastern Ethiopia where the prevalence of anemia among adolescent girls was 32% [6].

Increased family size may adversely affect the nutritional status of every member of the household, including adolescent girls, because it may be associated with decreased per capita human inputs. In other words, the allocation of food per household is likely to decrease with the increase in the family size, which, in turn, may adversely affect the nutritional status of adolescent girls. In line with this, the current study revealed that adolescent girls from family size ≥5 were approximately two times more likely to be anaemic [(AOR = 1.80), CI:(1.14, 2.85)] compared to those who from ≤5 people.

A survey study in Misamis oriental province has shown that reducing family size can be more effective in preventing nutritional problems among the high-risk group (pregnant women, children, and adolescent girls). The results indicate that decreases in family size will effectively improve the household’s level of living and the expected sequence of influence may be that: first, a decrease in the number of family size makes it more possible to buy adequate food; second, these foods have enough nutrient content that they meet the daily recommended dietary requirements; and third, when adequate nutritious foods are available, the good health of a family is improved [7]^.^in line with this, the current study revealed s that adolescent girls from family size ≥5 people were approximately two times more likely to be anaemic [(AOR = 1.80), CI: (1.14, 2.85)] when we compared those who from l family that are < 5 people.

Somali region approximately 100% is exposed to Malaria (World Bank, (2006). Malaria has a range of manifestations but malaria-related anemia is one of the leading causes of death, with reproductive women and children being the most affected [8]. Malaria infection causes anemia either in the early stage of infection, rupture of parasitized red blood or hypersplenism that result in clearance of both mature and not matured red blood cell [9], however, the current study doesn’t show a significant association b/n malaria and haemoglobin concentration of adolescent girls. The First reason, malaria infection was based on verbal history from participants rather than any test of blood and this might have masked the actual status of the respondents. The second reason, Somali region is a stable endemic malaria area (transmission of infection throughout the year) adolescent girls might develop high immunity.

Anemia was significantly associated level of knowledge of anemia among adolescent girls in this study. Adolescent girls who never heard anemia were 1.6 more likely to be anemic [AOR = 1.62), CI (1.01, 2.59)] compared to those who heard anemia. The possible reason for the high prevalence of anemia among students who never heard/know anemia could be poor knowledge regarding iron-rich foods compared to girls with knowledge about anemia that consider the prevention and control mechanism of anemia.

This is similar to observation done in Tatah Makmur South Kalimantan Public Health Center by Tumanggor, & Tumanggor (2017), who reported that the incidence of anemia was significantly associated level of knowledge of anemia among adolescent girls.

Menstruation is a monthly endometrial shedding leading to the discharge of blood from the uterus occurring every 8 ± 7 days and a part of the normal reproductive cycle of the female. The average menstrual bleeding lasts about 5 days [17]. It is known that heavy as well as menstrual bleeding for a prolonged period can lead to anaemia [10]. The current study revealed that 62.6% of adolescent girls had menstrual duration ≥5 days and 84.9% of adolescent girls had irregular menstruation patterns. However, this study indicated that both irregular menstruation patterns and duration menstruation ≥5 days were not significant to the haemoglobin concentration of adolescent girls. The possible reason behind more girls with an irregular cycle in our study could be due to the higher percentage of young girls aged, as the study suggested that normal cycle length is obtained around the chronological age of 19–20 [11]. Research results in line with our result is conducted in Depok City Region reported there is no significant relationship between the pattern of menstruation and the incidence of anaemia in adolescent girls [12].

### Strength

Hemoglobin (Hb) measurement used the recommended laboratory equipment (Hem cue HB 301 Analyzer) and procedures by trained laboratory technicians. The quality control issues were strictly followed as per the manual (guidelines). One-day intensive training was given for data collectors and supervisors. But this study might have some limitations; such only haemoglobin estimation was done other hematological parameters ware not estimated and malaria infection was based on recall history in the last 14 days and not laboratory-based which might compromise the accuracy of the data.

## Conclusion

Prevalence of anemia among adolescent’s school girls in Godey and Degehabur council Somali region was 31.5%. Family size ≥5 people and lack of anaemia knowledge were independent predictors of anemia. Therefore, the present study emphasizes the need for the development of health and nutrition education strategies to improve dietary habits, family planning, and intermittent iron-folic acid supplementation with intensifying adolescent anemia prophylaxis programs.

## Data Availability

All data generated or analyzed during this study are included in this published article.
